# The effect of a locally tailored intervention on the uptake of preconception care in the Netherlands: a stepped-wedge cluster randomized trial (APROPOS-II study)

**DOI:** 10.1186/s12889-022-14343-x

**Published:** 2022-11-01

**Authors:** V. Y. F. Maas, M. Poels, E. Ista, L. F. Menge, K. L. H. E. Vanden Auweele, R. W. A. de Bie, D. J. de Smit, E. H. van Vliet-Lachotzki, A. Franx, M. P. H. Koster

**Affiliations:** 1 Department of Obstetrics and Gynaecology, Erasmus MC, University Medical Centre Rotterdam, Doctor Molewaterplein 40, 3015 GD Rotterdam, The Netherlands; 2 House of Women, Niasstraat 7, 1095 TS Amsterdam, The Netherlands; 3 Department of Internal Medicine - Nursing Science, Erasmus MC, University Medical Centre Rotterdam, Doctor Molewaterplein 40, 3015 GD Rotterdam, The Netherlands; 4 Department of Paediatric Surgery, Paediatric intensive care, Erasmus MC Sophia Children’s Hospital, University Medical Centre Rotterdam, Doctor Molewaterplein 40, 3015 GD Rotterdam, The Netherlands; 5 Dutch Hellp Foundation (Hellp Stichting), Postbus 40126, 8004 DC Zwolle, The Netherlands; 6 Dutch Ministry of Health, Welfare and Sports, Parnassusplein 5, 2511 VX The Hague, The Netherlands; 7 Department of Obstetrics, Diakonessenhuis Hospital, Bosboomstraat 1, Utrecht, 3582 KE The Netherlands; 8 MediClara Projects, Prinses Beatrixstraat 7, 1396 KD Baambrugge, The Netherlands; 9 Dutch Genetic Alliance, VSOP, Koninginnelaan 23, 3762 DA Soest, The Netherlands

**Keywords:** Preconception care, Social marketing, Lifestyle Behaviour, Health promotion

## Abstract

**Background:**

The preconception period provides a window of opportunity for interventions aiming to reduce unhealthy lifestyle behaviours and their negative effect on pregnancy outcomes. This study aimed to assess the effectiveness of a locally tailored preconception care (PCC) intervention in a hybrid-II effectiveness implementation design.

**Methods:**

A stepped-wedge cluster randomized controlled trial was performed in four Dutch municipalities. The intervention contained a social marketing strategy aiming to improve the uptake (prospective parents) and the provision (healthcare providers) of PCC. Prospective parents participated by administering a questionnaire in early pregnancy recalling their preconceptional behaviours. Experiences of healthcare providers were also evaluated through questionnaires. The composite primary outcome was adherence to at least three out of four preconceptional lifestyle recommendations (early initiation of folic acid supplements, healthy nutrition, no smoking or alcohol use). Secondary outcomes were preconceptional lifestyle behaviour change, (online) reach of the intervention and improved knowledge among healthcare providers.

**Results:**

A total of 850 women and 154 men participated in the control phase and 213 women and 39 men in the intervention phase. The composite primary outcome significantly improved among women participating in the municipality where the reach of the intervention was highest (Relative Risk (RR) 1.57 (95% Confidence Interval (CI) 1.11–2.22). Among women, vegetable intake had significantly improved in the intervention phase (RR 1.82 (95%CI 1.14–2.91)). The aimed online reach- and engagement rate of the intervention was achieved most of the time. Also, after the intervention, more healthcare providers were aware of PCC-risk factors (54.5% vs. 47.7%; *p* = 0.040) and more healthcare providers considered it easier to start a conversation about PCC (75.0% vs. 47.9%; *p* = 0.030).

**Conclusion:**

The intervention showed some tentative positive effects on lifestyle behaviours among prospective parents. Primarily on vegetable intake and the knowledge and competence of healthcare providers. The results of this study contribute to the evidence regarding successfully implementing PCC-interventions to optimize the health of prospective parents and future generations.

**Trial registration:**

Dutch Trial Register: NL7784 (Registered 06/06/2019).

**Supplementary Information:**

The online version contains supplementary material available at 10.1186/s12889-022-14343-x.

## Background

Worldwide, every 11 seconds a pregnant woman or newborn dies, mostly due to preventable causes [1]. In 2008, it was reported that perinatal mortality and morbidity rates in the Netherlands were relatively high compared to other European countries. Since then, many health promotion initiatives have been launched focussing on the prevention and recognition of potential risk factors for adverse pregnancy outcomes, such as preterm birth and fetal growth restriction [2, 3]. Analysis of the collected evidence has shown that exposure to unhealthy lifestyle behaviours before or during pregnancy, such as smoking, unhealthy nutrition, alcohol consumption and physical inactivity, negatively affect pregnancy outcomes [4–6]. In addition, other studies suggest that women suffering from adverse pregnancy outcomes have an increased risk for developing long-term health issues, such as obesity, type 2 diabetes and cardiovascular- and respiratory disease [7–10]. Despite major advances in clinical research, medical technology and health promotion initiatives, only a moderate decrease in adverse pregnancy outcome rates has been observed in recent years [11, 12].

While over 85% of pregnancies in the Netherlands are planned, many women do not actively prepare for pregnancy by making recommended lifestyle adjustments, such as timely folic acid supplement use [13]. Moreover, the majority of men also make no lifestyle adjustments to improve their health and fertility prior to conception [14, 15]. As the first months of pregnancy are essential for implantation, organogenesis and placental development, and considering the high prevalence of unhealthy lifestyle behaviours in the reproductive population, the preconception period provides a window of opportunity to intervene [4, 5, 16–23]. Hence, preconception care (PCC) is introduced as a set of interventions aiming to identify and enable informed decision-making to modify biomedical, behavioural, and (psycho) social risks to parental health and the health of their future child, through counselling, prevention and management [24]. Despite increasing international recognition of the potential health-promoting benefits of PCC, the uptake of PCC-consultations remains remarkably low [25–27]. Suggested reasons for the low uptake of PCC include difficulties with reaching the target population and low awareness of preconceptional risk factors among healthcare providers [28].

Previously developed PCC-interventions have had only limited success and mainly focused on individual behaviour change rather than social, structural, and environmental elements that can influence preconception health, such as the social setting or general PCC-knowledge [29]. Hence, suggestions are made to actively engage prospective parents in PCC by creating a social movement and promoting preconceptional health through social marketing campaigns [30, 31]. A previous locally tailored PCC-intervention promoting preconceptional health was implemented and evaluated in a feasibility study (APROPOS-I) conducted by our group in 2017, which led to improved preconceptional lifestyle behaviours and increased use of PCC among prospective parents [32]. In the current study (APROPOS-II), we enhanced this previous PCC-intervention by including a social marketing strategy and a larger study population [33, 34]. This study aims to assess the effectiveness of the APROPOS-II intervention in a hybrid-II effectiveness implementation design on (1) adequate preconceptional (lifestyle) behaviours among prospective parents and (2) provision of PCC and PCC-knowledge among healthcare providers.

## Methods

### Study design

In this stepped-wedge cluster randomized controlled trial (hybrid-II), the locally tailored PCC-intervention of the APROPOS-II study was implemented and evaluated in four municipalities (clusters) in the Netherlands (Amersfoort, Barneveld, Deventer and Zoetermeer). Randomization occurred at cluster-level instead of an individual-level since the entire target population (prospective parents in their reproductive lifespan) was exposed to the intervention due to its community-based approach. A detailed description of the study design of the APROPOS-II study has been published elsewhere [33]. The participating municipalities represent the four clusters and varied in size from approximately 60,000 to 160,000 inhabitants per municipality [35]. This study was registered in the Dutch Trial Register on 06/06/2019, under trial number NL7784.

**Fig. 1:**
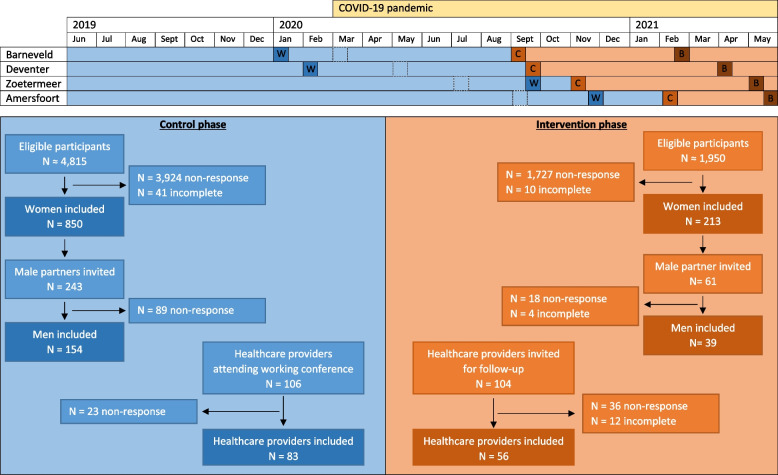
Timetable and flowchart of the participants Legend: W = working conference; C = campaign week; B = booster session; dashed line = originally planned campaign week but postponed due to the COVID-19 pandemic

The first registration of this study was obtained on 18/06/2019 and inclusion continued until 01/06/2021. According to our initial planning, implementation of the intervention in the first municipality should have taken place in March 2020. However, due to the COVID-19 pandemic, implementation was postponed and not launched until September 2020, the originally planned interventions are displayed in Fig. 1 with the dashed line. As a result, the intervention phase of the study was shorter than the control phase. This study was approved by the Medical Ethical Review Board (MEC-2019–0278) of the Erasmus MC, University Medical Centre Rotterdam and informed consent was obtained from all participants.

### Intervention

**Fig. 2:**
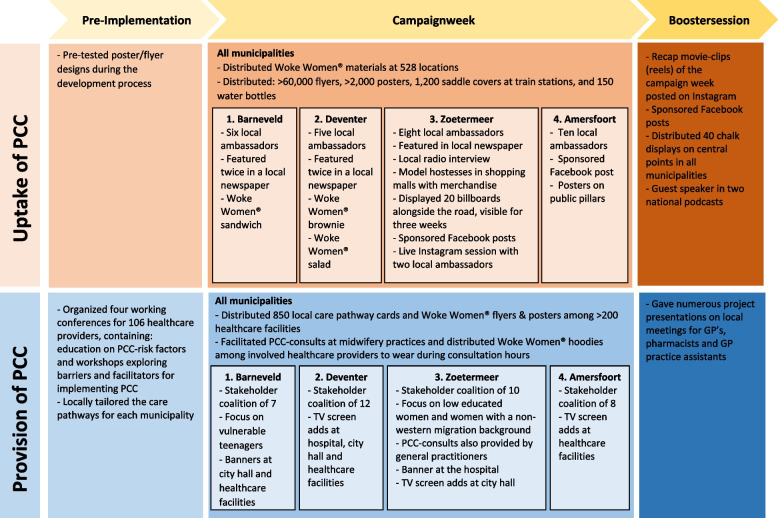
Overview of the intervention specifically developed for the APROPOS-II study

The PCC-intervention was specifically developed for this study and was based on pre-implementation research from the APROPOS feasibility study [32]. The intervention contained a dual-track approach and focused on the uptake of PCC (the prospective parents) as well as the provision of PCC (healthcare providers). A detailed overview of the content of the intervention is shown in Fig. 2 and described below:

#### Focus on uptake of PCC (prospective parents)

In collaboration with a marketing agency, a new social marketing strategy called “Woke Women®” was developed. This social marketing strategy uses commercial marketing technologies to improve preconceptional behaviours of the target population (prospective parents) [36]. The slogan of the Woke Women® campaign was: ‘Wake up smart (future) mama! Let’s make your baby strong’. A detailed description of the development of the Woke Women® campaign has previously been published [34]. During the entire intervention phase, the following implementation strategies were used: PCC-consultations were facilitated at all midwifery practices as part of standard care, but also evidence-based PCC-information was distributed using different mediums, such as posters, flyers, billboards, bicycle saddle covers, newspaper items, radio programs, word-of-mouth marketing and social media feeds. All these items were used to refer prospective parents to the project website (www.wokewomen.nl) containing evidence-based information on preconception health and risk factors. The intervention phase was initiated with a large campaign week during which the campaign was launched with lots of media attention. The target population was reached through several (social) media channels using organic feed, stories and give-away actions. Our Instagram (@Woke_Women_NL) and Facebook channels were promoted and endorsed by local ambassadors. Local ambassadors are influential women with broad networks within society (e.g., online influencers, local hairdressers, maternity fitness coaches and local restaurant owners) who act as potential role models for prospective parents within their community, and can inspire them to optimize their (preconceptional) health. The local ambassadors were an important element of the intervention, providing opportunities to collaborate with them on special promotions (e.g. Woke Women® sandwich and salad), events, social media and the offline distributing of campaign materials.

#### Focus on provision of PCC (healthcare providers)

In all participating municipalities, a working conference among healthcare providers of multidisciplinary backgrounds (e.g. midwives, general practitioners, dieticians, physiotherapists, etc.) was organized approximately three months prior to the intervention, to educate them on preconceptional health and risk factors and to conduct a region-specific bottleneck analysis [13]. Next, a smaller multidisciplinary group of healthcare providers formed a local stakeholder coalition to tailor the PCC-intervention to their municipality, e.g. listing distribution locations for the campaign materials, suggesting potential collaborating partners, and establishing a primary target population for the intervention. To improve interdisciplinary collaboration among healthcare providers, a local care pathway was developed and implemented in all municipalities, including interdisciplinary arrangements for collaboration and referral between primary, secondary and tertiary care.

### Participants and setting

During the two-year inclusion period, every pregnant woman who attended one of the eight recruiting community midwifery practices for their booking visit were asked by their midwife to participate in the study. These midwifery practices were all located in four municipalities (i.e. the clusters), with together approximately 3,400 booking visits per year. All pregnant women above the age of 18 years who adequately mastered the Dutch, English, Polish or Turkish language were eligible and approached to participate in the study. Prospective fathers were recruited through their participating partners and were also asked to participate in the study. Given the stepped-wedge design of the study, participants were enrolled in either the control- or intervention group depending on the time of inclusion.

At the end of each working conference, attending healthcare providers were asked to participate in the study by administering a questionnaire on their perceptions on the provision of PCC. The same healthcare providers, plus all midwives working in one of the recruiting midwifery practices, were asked to anonymously fill out a follow-up questionnaire three to six months after the intervention. Hence, the group of healthcare providers before and after the intervention were only partly identical.

### Data collection and outcome measures

#### Prospective parents

After providing informed consent, participation in the study required filling out a single questionnaire that took an average of 15 min to complete and could be administered online or via hardcopy. The questionnaire was available in Dutch, English, Polish and Turkish, languages mastered by the majority of the inhabitants of the participating municipalities. The questionnaire for prospective parents included aspects of previously validated questionnaires, such as the London Measure of Unplanned Pregnancy, the Dutch preconception tool ‘Preparing for Pregnancy’ (www.zwangerwijzer.nl) and the APROPOS feasibility study [32, 37, 38]. The questionnaire for women contained four main sections: pregnancy planning, pregnancy preparation, lifestyle behaviours and risk factors, and demographic characteristics. To assess preconceptional lifestyle behaviour change, several questions elaborated exactly on when initiation or cessation of these behaviours took place, either before or after pregnancy recognition. The questionnaire for men was shorter and solely focused on periconceptional lifestyle behaviours and pregnancy planning. For both the control- and the intervention group, the same questionnaire was used, except for six additional campaign evaluation questions in the intervention group. Questionnaires of participants who discontinued before finishing the first section (12% of the questionnaire), were regarded as incomplete questionnaires. The English versions of the questionnaires for women and men are provided in the Supplemental Material.

The primary outcome of this study among prospective parents was improved preconceptional lifestyle behaviour. This primary outcome was a dichotomous composite outcome expressed for women as adherence to at least three of four preconceptional lifestyle behaviour recommendations: 1) timely initiation of folic acid supplements (> 400 micrograms ≥ 4 weeks before conception), 2) no alcohol use, 3) no smoking, and 4) adherence to at least two of the following preconceptional nutrition recommendations: fruit intake (≥ 2 pieces per day), vegetable intake (≥ 250 g per day) and caffeine intake (≤ 2 beverages per day) [13, 39–41]. For men, the primary outcome was expressed as the adherence to two preconceptional lifestyle recommendations (not smoking nor using alcohol).

Secondary outcomes among prospective parents were:

- Change from inadequate to adequate lifestyle behaviours regarding preconceptional alcohol use and smoking;
- The preconceptional behaviours: retrieved PCC-information (e.g. internet, books, journals, folders or family & friends) and/or visited a PCC-consultation;
- Reach of the intervention: the offline and online reach of the intervention among the participants, was measured through 1) the questionnaire, in which participants were asked whether they took notice of the Woke Women® campaign after providing some examples of the distribution materials, and 2) visiting- and interaction statistics from the Woke Women website (Google analytics) and Instagram account (Instagram statistics). These latter were expressed as: the number of weekly website visits, number of Instagram followers per month, and number of views and reactions (likes/comments/shares) per Instagram post. The reach rate (the number of views divided by the number of followers) and the engagement rate (the number of reactions divided by the number of followers) were calculated per post and averaged per week. Based on similar Instagram accounts, we aimed for a reach rate of ≥ 36% and an engagement rate of ≥ 4.5%.

The following patient characteristics were assessed in both women and men: age, ethnic background (Dutch or non-Dutch), parity (first-time parent or no first-time parent), mode of conception (spontaneous or assisted reproduction) and educational level (low/moderate or high). According to Dutch classifications, low educational attainment contains: no education, lower vocational education and lower secondary education; moderate educational attainment contained: intermediate vocational education, high school, pre-university education; and high educational attainment contained: higher vocational education and university [42]. In the questionnaire for women, we also assessed the presence of a chronic illness, e.g. asthma or diabetes (yes or no) and pre-pregnancy weight and height. Hence, Body Mass Index (BMI) was calculated and categorised as underweight (< 18.5 kg/m2), normal weight (18.5–24.9 kg/m2), overweight (25.0–29.9 kg/m2) and obese (≥ 30.0 kg/m2) [43]. While originally planned in the protocol paper, due to logistical constraints as a result of the COVID-19 pandemic pregnancy- and neonatal outcomes were not collected in this study. In addition, results on the relation between pregnancy planning (using the London Measure of Unplanned pregnancy), health beliefs and preconceptional lifestyle behaviours with data of the control phase, has recently been published elsewhere [44].

#### Healthcare providers

The questionnaire for healthcare providers assessed how PCC impacted their daily work routine and contained eight validated Normalisation MeAsure Development (NOMAD) statements and two additional statements on healthcare providers’ beliefs regarding PCC [45, 46]. We hypothesized that the PCC-intervention could impact the work of midwives differently than of non-midwives, since non-midwives are usually not trained to provide PCC. Hence, the questionnaire for non-midwives contained three additional statements. Agreement to the statements were assessed either on a ten-point scale (1 = not at all – 10 = completely) or on a five-point scale (1 = disagree – 5 = agree). The English version of the questionnaires for healthcare providers is provided in the Supplemental Material.

The main outcome measures for healthcare providers were:

- Provision of PCC: the yearly number of PCC-consultations provided before and after the intervention.
- PCC-beliefs: based on answers to the statements regarding PCC in the questionnaire before and after the intervention.

The following characteristics were assessed among healthcare providers: profession, municipality, years of working experience in their current job (< 5 years – 5–20 years – > 20 years) and the yearly number of PCC-consultations provided (never – 1–4 times per year – ≥ 5 times per year).

### Sample size and data analysis

In accordance with the sample size estimation method of Hemming and Taljaard, the sample size for this study was determined on 2,167 female participants (initially based on six municipalities) [47]. Baseline data for all participants are presented as numbers and percentages for categorical variables or as medians and interquartile ranges (IQR) for continuous variables. In accordance with the study design of a randomized controlled trial, we identified differences in adherence and change of preconceptional (lifestyle) behaviours between the control- and intervention group, relative risks (RR) with 95% confidential intervals (CI). Risks were calculated for the total group as well as per municipality. Due to the low sample size, as a result of the COVID-19 pandemic, we opted to refrain from the ANCOVA-test as originally planned in our protocol. Differences in awareness of the campaign per municipality were assessed with a chi-square test.

To assess differences in baseline characteristics between healthcare providers in the control- and intervention group, chi-square tests for categorical variables and Mann–Whitney U-tests for continuous variables were performed. For the analysis, the five-point scales answer options of the statements were adjusted to three-point scales (disagree, neutral or agree). Differences in responses between healthcare providers in the control- and intervention phase were compared using chi-square tests (for the statements with a three-point scale) or Mann–Whitney U-tests (for the statements with a ten-point scale). All data were analysed using IBM SPSS for Windows version 25.0 and *P*-values < 0.05 were considered statistically significant.

## Results

### Baseline characteristics of prospective parents

A total of 1,063 pregnant women participated in our study, of which 850 (80.0%) enrolled during the control phase and 213 (20.0%) during the intervention phase (Fig. 1). In addition, 193 male partners enrolled in the study; 154 (79.8%) in the control phase and 39 (20.2%) in the intervention phase. The median age was 31 years (IQR = 28–33) for the participating women and 33 years (IQR = 30–36) for the participating men. The majority of the participating prospective parents were of Dutch origin (960 women, 94.5%; 191 men, 99.0%) and highly educated (658 women, 61.9%; 128 men, 66.7%). All sociodemographic characteristics between the control and intervention groups were comparable, except for parity. The intervention group represented more first-time parents, 58.5% versus (vs) 45.6% among women and 69.2% vs 51.3% among men (Table 1).

**Table 1:** Baseline characteristics of participants in the control- and intervention-phase

	**Women**		**Men**	
	Control	Intervention	Control	Intervention
	*n* = 850	*n* = 213	*n* = 154	*n* = 39
**Age (years)**^a^	31.0 (28.0–33.0)	31.0 (28.0–34.0)	33.0 (30.0–36.0)	33.0 (30.0–35.0)
< 25	43 (5.3)	21 (10.1)	8 (5.2)	2 (5.1)
25—29	266 (32.9)	58 (28.0)	20 (13.0)	5 (12.8)
30—34	367 (45.4)	87 (42.0)	73 (47.4)	20 (51.3)
35—39	117 (14.5)	32 (15.5)	40 (26.0)	6 (15.4)
≥ 40	16 (2.0)	9 (4.4)	13 (8.4)	6 (15.4)
*Missing data*	*41*	*6*		
**Ethnical background**				
Dutch	768 (94.9)	192 (92.8)	152 (98.8)	39 (100.0)
Non-Dutch	41 (5.1)	15 (7.2)	2 (1.2)	0 (0.0)
*Missing data*	*41*	*6*		
**Educational Level**				
Low/Moderate	288 (35.7)	68 (32.9)	47 (30.7)	17 (43.6)
High	519 (64.3)	139 (67.2)	106 (69.3)	22 (56.4)
*Missing data*	*43*	*6*	*1*	
**Pre-pregnancy BMI**^a^	23.5 (21.5–27.1)	23.7 (21.6–26.4)		
< 18.5	13 (1.6)	3 (1.4)		
18.5 – 24.9	499 (62.1)	124 (60.0)		
25.0 – 29.9	177 (22.0)	46 (22.2)		
≥ 30.0	115 (14.3)	34 (16.4)		
*Missing data*	*46*	*6*		
**Chronic illness**				
Yes	110 (13.6)	32 (15.2)		
No	698 (86.4)	178 (84.8)		
*Missing data*	*42*	*3*		
**First time parent**				
Yes	369 (45.6)	121 (58.5)	79 (51.3)	27 (69.2)
No	440 (54.4)	86 (41.5)	75 (48.7)	12 (30.8)
*Missing data*	*41*	*6*		
**Spontaneous conception**				
Yes	762 (93.2)	188 (93.1)	143 (93.5)	38 (97.4)
No	56 (6.8)	14 (6.9)	10 (6.5)	1 (2.6)
*Missing data*	*32*	*11*	*1*	

Data is presented as N(%)

^a^Median (IQR)

### Preconceptional (lifestyle) behaviours

The primary outcome (adherence to preconceptional lifestyle recommendations) showed no statistical difference between the control- and intervention-phase among women (RR 1.05; 95% CI 0.87–1.27) (Table 2). Overall, more women in the intervention phase adhered to the recommended preconceptional lifestyle behaviours compared to the women in the control phase, but none of these differences were statistically significant, except for vegetable intake (RR 1.82; 95% CI 1.14–2.91). Women tended to be more likely to preconceptionally quit alcohol use (RR 1.09; 95% CI 0.80–1.50) and smoking (RR 1.30; 95% CI 0.44–3.81) in the intervention phase compared to the control phase, although this was not statistically significant. Women in the intervention phase also tended to be more likely to retrieve PCC-information (RR 1.05; 95% CI 0.93–1.18) or visit a healthcare provider for a PCC-consultation (RR 1.15; 95% CI 0.90–1.48) compared to women in the control phase, although again this was not statistically significant.

More men in the intervention phase adhered to both preconceptional lifestyle recommendations (no preconceptional smoking nor alcohol use; RR 1.07; 95% CI 0.65–1.77), compared to men in the control phase. Also, in the intervention phase, more men made ceased their alcohol consumption before conception compared to men in the control phase (RR 1.71; 95% CI 0.64–4.55). Again, both these differences showed no statistical significance.

**Table 2:** Preconceptional (lifestyle) behaviours before and after exposure to the PCC-intervention

	**Women**			**Men**		
	Control	Intervention	RR (CI)	Control	Intervention	RR (CI)
	*n* = 850	*n* = 213		*n* = 154	*n* = 39	
**Retrieved PCC-Information**						
Yes	496 (60.6)	132 (63.5)	1.05 (0.93–1.18)	68 (46.6)	14 (37.8)	0.81 (0.52–1.27)
No	323 (39.4)	76 (36.5)		78 (53.4)	23 (62.2)	
*Missing data*	*31*	*5*		*8*	*2*	
**Visited PCC-Consultation**						
Yes	204 (24.5)	60 (28.3)	1.15 (0.90–1.48)	36 (23.4)	7 (17.9)	0.77 (0.37–1.59)
No	628 (75.5)	152 (71.7)		118 (76.6)	32 (82.1)	
*Missing data*	*18*	*1*		*0*	*0*	
**Fruit intake**						
Adequate (≥ 2 pieces)	203 (25.0)	54 (25.7)	1.03 (0.79–1.33)			
Inadequate (< 2 pieces)	610 (75.0)	156 (74.3)				
*Missing data*	*37*	*3*				
**Vegetable intake**						
Adequate (≥ 250 grams)	49 (6.0)	23 (11.0)	**1.82 (1.14–2.91)**			
Inadequate (< 250 grams)	764 (94.0)	187 (89.0)				
*Missing data*	*37*	*3*				
**Caffeine intake**						
Adequate (≤ 2 beverages)	403 (49.6)	98 (46.7)	0.94 (0.80–1.11)			
Inadequate (> 2 beverages)	410 (50.4)	112 (53.3)				
*Missing data*	*37*	*3*				
**Preconceptional nutrition**^a^						
≥ 2 adequate nutrition intakes	119 (14.6)	38 (18.1)	1.24 (0.89–1.72)			
< 2 adequate nutrition intakes	694 (85.4)	172 (81.9)				
*Missing data*	*37*	*3*				
**Folic acid supplements**						
Started in time (≥ 4 weeks before conception)	482 (59.5)	121 (57.6)	0.97 (0.85–1.10)			
Started too late or never	328 (40.5)	89 (42.4)				
*Missing data*	*40*	*3*				
**Alcohol use**						
Adequate (no preconceptional alcohol use)	415 (51.3)	108 (52.7)	1.03 (0.89–1.19)	52 (33.8)	14 (35.9)	1.06 (0.66–1.71)
Inadequate (used alcohol after conception)	394 (48.7)	97 (47.3)		102 (66.2)	25 (64.1)	
*Missing data*	*41*	*8*		*0*	*0*	
**Change in alcohol use**^b^						
Quit before conception	130 (24.8)	36 (27.1)	1.09 (0.80–1.50)	11 (9.7)	5 (16.7)	1.71 (0.64–4.55)
Quit after conception / still uses alcohol	394 (75.2)	97 (72.9)		102 (90.3)	25 (83.3)	
*Missing data*	*326*	*80*		*41*	*9*	
**Smoking**						
Adequate (no preconceptional smoking)	712 (87.7)	183 (87.1)	0.99 (0.94–1.05)	142 (92.2)	35 (89.7)	0.97 (0.88–1.09)
Inadequate (smoked after conception)	100 (12.3)	27 (12.9)		12 (7.8)	4 (10.3)	
*Missing data*	*38*	*3*		*0*	*0*	
**Change in smoking**^b^						
Quit before conception	11 (9.9)	4 (12.9)	1.30 (0.44–3.81)	8 (40.0)	4 (50.0)	1.25 (0.52–3.00)
Quit after conception / still smokes	100 (90.1)	27 (87.1)		12 (60.0)	4 (50.0)	
*Missing data*	*739*	*128*		*134*	*31*	
**Adherence to lifestyle recommendations**^c^						
Adequate	305 (37.5)	83 (39.5)	1.05 (0.87–1.27)	48 (31.2)	13 (33.3)	1.07 (0.65–1.77)
Inadequate	508 (62.5)	127 (60.5)		106 (68.8)	26 (66.7)	
*Missing data*	*37*	*3*		*0*	*0*	

Data is presented as N(%)

^a^ Combination of: fruit-, vegetable- and, caffeine intake

^b^ Of the participants preconceptionally using alcohol or smoking

^c^ For women, adherence to at least three of the following lifestyle recommendations: started folic acid supplements in time, ≥ 2 adequate nutrition intakes and no smoking nor alcohol use before conception

For men, adherence to both lifestyle recommendations: no smoking nor alcohol use before conception

### Reach of the Woke Women® campaign

During the campaign weeks, over 60,000 flyers and 2,000 posters were distributed among 528 locations in all four municipalities (Fig. 2). Of all the women participating in the intervention phase, 34 (16.3%) women could actively recall noticing the Woke Women® campaign. Social media activities (53.0%) and the distribution of flyer materials (17.7%) were the most recalled campaign items. Awareness of the campaign significantly differed per municipality (*p* < 0.001), varying from 2.9% of women actively recalling the campaign in Zoetermeer to 32.5% in Deventer (Table 3). In municipalities where the campaign was more frequently noticed, women also retrieved PCC-information and more often adhered to preconceptional lifestyle recommendations compared to municipalities with a lower awareness. For example, in Amersfoort, 32.0% of women in the intervention phase noticed the campaign and significantly more women adhered to at least three lifestyle recommendations after the intervention (RR 1.57 (95% CI 1.11–2.22)) (Table 3).

**Table 3:** Effect of the intervention among women stratified per municipality

	**Barneveld**			**Deventer**			**Zoetermeer**			**Amersfoort**		
	Control 104	Int 40	RR (CI)	Control 304	Int 80	RR (CI)	Control 268	Int 68	RR (CI)	Control 174	Int 25	RR (CI)
Noticed the campaign (reach)*		13 (32.5)			11 (13.8)			2 (2.9)			8 (32.0)	
**Retrieved PCC-Information**												
Yes	39 (39.8)	19 (51.4)	1.30 (0.87–1.30)	185 (63.4)	53 (66.2)	1.05 (0.87–1.25)	172 (65.9)	42 (63.6)	0.97 (0.79–1.18)	100 (59.5)	18 (72.0)	1.21 (0.92–1.59)
No	59 (62.2)	18 (48.6)		107 (36.6)	27 (33.8)		89 (34.1)	24 (36.4)		68 (40.5)	7 (28.0)	
Missing	6	3		12			7	2		6		
**Visited PCC-Consultation**												
Yes	14 (13.9)	6 (15.0)	1.08 (0.45–2.62)	85 (28.5)	28 (35.4)	1.24 (0.88–1.76)	57 (21.9)	18 (26.5)	1.21 (0.76–1.90)	48 (27.7)	8 (32.0)	1.15 (0.62–2.15)
No	87 (86.1)	34 (85.0)		213 (71.5)	51 (64.6)		203 (78.1)	50 (73.5)		125 (72.3)	17 (68.0)	
Missing	3	0		6	1		8	0		1		
**Adherence to lifestyle recommendations**^a^												
Adequate (≥ 3 recommendations)	26 (26.5)	15 (39.5)	1.49 (0.89–2.49)	109 (37.6)	31 (39.2)	1.04 (0.76–1.43)	100 (39.5)	21 (30.9)	0.78 (1.15–0.53)	70 (40.7)	16 (64.0)	**1.57 (1.11–2.22)**
Inadequate (to < 3 recommendations)	72 (73.5)	23 (60.5)		181 (62.4)	48 (60.8)		153 (60.5)	47 (69.1)		102 (59.3)	9 (36.0)	
*Missing data*	6	2		14	1		15	0		2	0	

Data is presented as N(%)

*Int.* Intervention

^a^ Adherence to at least three of the following lifestyle recommendations: started folic acid supplements in time, ≥ 2 adequate nutrition intakes and no smoking nor alcohol use before conception

^*^ Significant difference with the chi-square test, *p*-value < 0.001

**Fig. 3:**
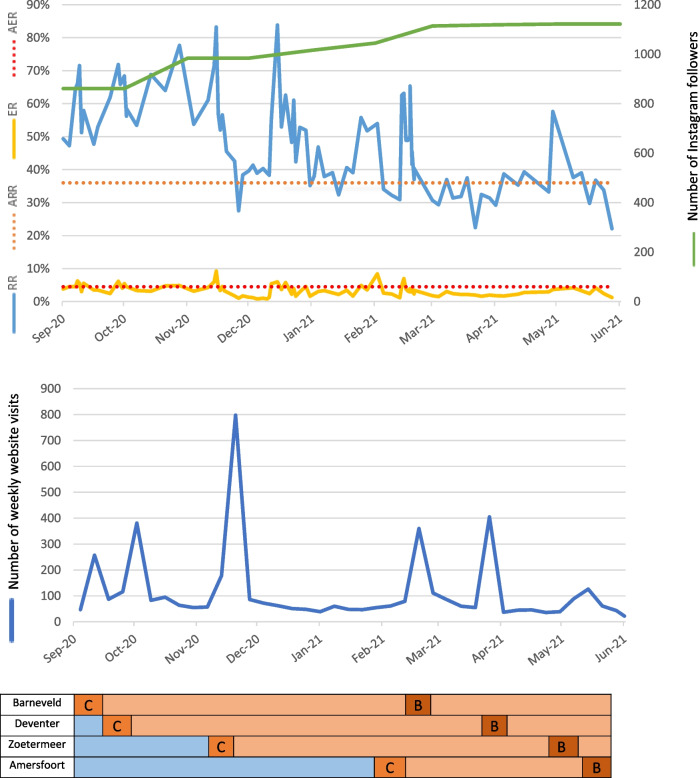
The effect of the intervention on reaching the target population online during the intervention period Legend: AER = Aimed Engagement Rate; ARR = Aimed Reach Rate; ER = Engagement Rate; RR = Reach Rate; C = campaign week; B = booster session

Online data derived from the Woke Women® website and Instagram account are visualised in Fig. 3. During the intervention period of nine months, the number of Instagram followers rose to 1,122 and a total of 100 online posts with PCC-information were distributed. The aimed reach rate of ≥ 36% was achieved most of the time, varying from 22.1% to 83.8%. The aimed engagement rate of ≥ 4.5% was achieved intermittently, varying from 0.8% to 9.3%. The project website was visited over 5,300 times during the intervention phase with an average of 114 visits per week. During the campaign weeks, the number of website visits increased by almost 700% up to 800 visits in one week.

### Healthcare providers

**Fig. 4:**
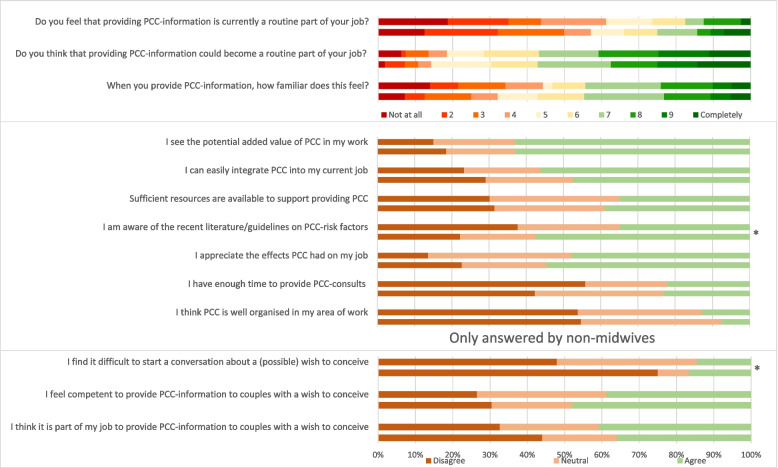
Effect of the intervention among healthcare providers Legend: Upper bars represent control group, lower bars intervention group; * Significant difference

A total of 83 healthcare providers participated in the control phase (78% response rate) and 56 in the intervention phase (53% response rate) (Table 4). The majority of healthcare providers were midwives, followed by healthcare providers working at preventive child health services. The number of provided PCC-consultations was comparable before and after the intervention (*p* = 0.760). In the intervention phase, healthcare providers were significantly more often aware of the recent literature/guidelines on PCC-risk factors (54.5% vs 47.7%; *p* = 0.040) compared to the control phase (Fig. 4). In addition, in the intervention phase, non-midwives felt more competent to provide PCC-information to couples with a wish to conceive (47.8% vs 38.8%; *p* = 0.534) and found it significantly easier to start a conversation about a possible wish to conceive (75.0% vs 47.9%; *p* = 0.030) compared to the control phase.

**Table 4:** Baseline characteristics of healthcare providers

	**Control** *N* = 83	**Intervention** *N* = 56	*P*-Value^1^
**Profession**			
Midwife	30 (36.1)	29 (51.8)	**0.041**
Preventative Child Health Services	14 (16.9)	8 (14.3)	
Dietician	8 (9.6)	5 (8.9)	
Physiotherapist	7 (8.4)	5 (8.9)	
General Practitioner	5 (6.0)	2 (3.6)	
Gynaecologist	3 (3.6)	1 (1.8)	
Other	16 (19.4)	6 (10.7)	
**Municipality**			
1. Barneveld	20 (24.1)	18 (32.1)	0.717
2. Deventer	31 (37.3)	17 (30.4)	
3. Zoetermeer	18 (21.7)	11 (19.6)	
4. Amersfoort	14 (16.9)	10 (17.9)	
**Working experience**^a^	11.5 (5.0–24.3)	15.0 (6.4–24.5)	0.265
< 5 years	19 (23.2)	9 (16.1)	
5—20 years	35 (42.7)	25 (44.6)	
> 20 years	28 (34.1)	22 (39.3)	
Missing data	1		
**Provides PCC-consultations**			
Never	33 (42.9)	22 (40.0)	0.760
1—4 times/year	26 (33.8)	17 (30.9)	
≥ 5 times/year	18 (23.4)	16 (29.1)	
Missing data	6	1	

Data is presented as N(%)

^a^ Median (IQR)

^1^ *P*-value for categorical variables (chi-squared test) and for the continuous variable (Mann–Whitney U test)

## Discussion

### Main findings

This study presents the effectiveness of the implementation of a locally tailored PCC-intervention in four municipalities in the Netherlands. We showed some tentative positive effects since pregnant women in the intervention phase were more prone to adhere to preconceptional lifestyle recommendations, especially among women from municipalities in which the awareness of the campaign was higher. While the increased website visits in the intervention phase were closely associated with the timing of the campaign weeks, social media showed to be a more consistent medium to reach the target population in all municipalities simultaneously. Finally, in the intervention phase, healthcare providers were more aware of the recent literature/guidelines on PCC-risk factors and experienced fewer barriers to start a conversation about a possible wish to conceive, compared to healthcare providers in the control phase.

### Interpretation

The results of our study show that, despite extensive efforts and a multifaceted intervention, it remains difficult to reach the target population (i.e. women and men of reproductive age) since only 16% of the participants could actively recall the intervention. Previous health awareness campaigns in the field of maternity care, varying in size of the targeted region and extent of the intervention, showed recall rates varying between 12 and 55% [32, 48, 49]. One of the biggest challenges in developing a successful social marketing strategy for PCC is finding one single strategy that resonates with a large and diverse target population [50]. A previous study suggested developing different strategies for different target populations: (1) the general reproductive-aged population; (2) women and men planning to conceive; and (3) high-risk groups, including women and men in difficult social situations, those who have had previous adverse pregnancy outcomes, and those with known medical disorders [24]. We incorporated these strategies in our intervention for instance by recruiting local ambassadors with a network amongst several high-risk populations, since previous literature showed that these groups are preferably reached through their social network [51]. Other previously suggested difficulties when implementing a social marketing strategy for PCC entail that PCC is an unknown form of care, it assumes that pregnancies are always planned, prospective parents have a desire to keep their wish to conceive secret, and that there is a diverse set of behaviours involved in PCC [50, 52–57].

Our locally tailored PCC-intervention showed only a limited effect on preconceptional lifestyle behaviour adherence and change compared to previously developed PCC-interventions focusing on implementing individualized PCC-counseling, demonstrating improved daily pre-pregnancy multivitamin consumption, healthier diet and alcohol cessation before pregnancy [17, 18, 32]. Presumably, a PCC-intervention using a community approach requires more time to become effective. Hence, changing health behaviours, in general, is difficult, time consuming and commonly underestimated [58]. Therefore, as previous research already advocated and the results of our study reconfirm, health campaigns can have important functions and can be effective, however, they should be part of larger (programmatic) multifaceted interventions focussing on different individual approaches since behaviour change is not just about basic messaging [58, 59]. Here lies an opportunity to distribute information through social media and local ambassadors/influencers, since these mediums are valued for their potential to engage with the target audience to promote programs, products, and services [60–62].

With regard to healthcare providers, previous research showed that even though the majority of professionals have positive attitudes towards PCC, knowledge on PCC-risk factors is lacking and there is a need for education and postgraduate courses [46, 63–65]. The healthcare providers that participated in our study reported improved knowledge on the recent literature/guidelines on PCC-risk factors after the intervention, proving how our PCC-intervention is a successful tool to increase such PCC-knowledge. However, still, the majority of healthcare providers did not agree with the statement that PCC is well organised in their municipality. Therefore, based on our study results and recommendations made in previous studies, we suggest structurally embedding PCC in a healthcare system and sharing accountability and responsibility for providing PCC [46, 66, 67]. Increasing awareness among multidisciplinary healthcare professionals and the target population on the potential health benefits that PCC can generate is a potential first step. Hence, a shift is needed towards a healthcare system in which childbearing plans and reproductive health are actively addressed to every couple, every time.

### Strengths and limitations

To our knowledge, this was the first randomized study assessing the effectiveness of an evidence-based social marketing strategy for PCC on preconceptional lifestyle recommendation adherence. One of the strengths of this study is its dual-track approach, i.e. focussing on both the uptake (among prospective parents) and provision (among healthcare providers) of PCC, thereby collecting data from different points of view. In addition, this study is one of the first studies to measure the effect of a PCC-intervention through online data derived from website visits as well as social media data regarding reach rate and engagement rate.

The COVID-19 pandemic has had a large impact on our study; not only did we had to postpone the launch of the campaign weeks for a minimum of six months, but the lockdown measures also caused many people to stay at home resulting in impaired visibility of the offline part of the campaign. We originally planned to implement the intervention in six different municipalities in the Netherlands, however, after the start of the study the first municipality withdrew from participating due to logistical reasons and launching the campaign in the final municipality was no longer feasible due to a second COVID-19 lockdown. All this has resulted in a smaller sample size than expected and a size difference between the control- and intervention phases, possibly affecting the scope of our study design due to the COVID-19 pandemic. The uneven distribution of inclusions between the control- and intervention phases might also be explained by the differences in size between municipalities. This small sample size may have impeded us from adequately measuring the effects of the intervention on the primary outcome (i.e. adherence to preconceptional lifestyle recommendations). Nonetheless, the sample size of this cohort still exceeds many previous national interventions attempting to improve preconceptional behaviours [32, 68–70]. In addition, due to the low sample size, we were not able to perform an in-depth analysis of the differences between clusters. Therefore, clustering bias could have occurred. However, since the comparability of the participants’ characteristics between the control- and invention group is high, we don’t expect this potential bias to have affected our results too much. Another limitation of this study is potential responder bias. While in the control phase still 78% of healthcare providers who attended the working conference participated in the study, in the intervention phase this rate decreased to 54%, possibly indicating the lack of interest in the topic. Among prospective parents this trend was also visible; from a 18% response rate in the control phase to a 11% response rate in the intervention phase (Fig. 1). Together with an overrepresentation of highly educated Dutch women, this may potentially affect the generalisability of our study’s results. A population-effect might have occurred. Future studies should focus on follow-up research within a more heterogeneous study population. Finally, the design of our study could have hindered us to evaluate the total coverage of the intervention. Since it is very difficult to reach women who are actively preparing for pregnancy as they tend to keep their wish to conceive secret, we chose to measure the effect of the intervention solely among prospective parents who successfully conceived [28]. By doing so, we missed the opportunity to measure the intervention’s effect among prospective parents who were trying to conceive, but are not yet pregnant.

### Future perspectives

The results of this study show that reaching the target population to provide PCC-information and to encourage them to change their lifestyle behaviour remains difficult. We recommend that future studies focus on reaching the target population within diverse settings using various channels since there is no one-size-fits all solution. Future studies could, therefore, expand these approaches to develop more sustainable PCC-intervention to provide better preventive care to future parents. An extensive process evaluation of our study analyzing the differences between clusters could provide insights into how social marketing strategies resonate among different target populations. To emphasize the urgency to invest in PCC-programs, future research is suggested to include a broad set of (pregnancy) outcomes and (non-medical) preconceptional risk factors to assess the potential health gain of PCC, not solely measuring lifestyle behaviour but also evaluating awareness, knowledge and successful channels to reach the target population. Learnings from this study could contribute to the development of prospective PCC-interventions as it is suggested it takes years to change public opinion regarding the proposed normality to actively prepare for pregnancy and attain healthy preconceptional lifestyle behaviours [71].

## Conclusion

This study shows that a locally tailored PCC-intervention containing a social marketing strategy to encourage prospective parents to actively prepare for pregnancy has the potential to improve preconceptional lifestyle behaviours. The intervention showed some tentative positive effects on lifestyle behaviours among prospective parents, knowledge and competence among healthcare providers and reaching the target population online. However, the sample size in this study was small and health behaviour changes were marginal. Nevertheless, the results of this study contribute to the evidence regarding the barriers and facilitators for implementing PCC-interventions to optimize the health of prospective parents and future generations.

## Supplementary Information

**Additional file 1: Supplemental File 1.** Questionnaire for women. **Supplemental File 2.** Questionnaire for men. **Supplemental File 3.** Questionnaire for healthcare providers.

## Data Availability

The dataset for the current study is available from the corresponding author upon reasonable request.

## Abbreviations

BMI: Body mass index
CI: Confidence interval
IQR: Interquartile range
NoMAD: Normalisation MeAsure Development
PCC: Preconception care
RR: Relative risks
SPSS: Statistical Package for the Social Science
VS: Versus
