# Supplementary material for: The effect of a locally tailored intervention on the uptake of preconception care in the Netherlands: a stepped-wedge cluster randomized trial (APROPOS-II study)
Source: BMC Public Health. 2022 Nov 1;22:1997. doi: 10.1186/s12889-022-14343-x (PMC9623982; doi:10.1186/s12889-022-14343-x)
Supplement: Supplementary file 1 — Additional file 1: Supplemental File 1. Questionnaire for women. Supplemental File 2. Questionnaire for men. Supplemental File 3. Questionnaire for healthcare providers. [file 12889_2022_14343_MOESM1_ESM.zip › 20220622 - Supplemental file 1 - Questionnaire women.docx]

APROPOS-II Questionnaire pregnant women

Will you help us improve maternity healthcare in the period before pregnancy?

APROPOS-II
Project For a scientific study from Erasmus MC in Rotterdam we want to know how prospective parents prepare for the pregnancy and what these preparations have for consequences on the health of mothers and their babies. Your opinion and experiences are very important for us. Through a questionnaire we can investigate what your wishes, needs and experiences are regarding a healthy pregnancy. Through your participation we can improve the care for women who want to become pregnant in the future.

What is preconception care?
By preconception care we mean: all healthcare and information that you received before you became pregnant in order to prepare for your pregnancy as well as possible. Think of information on the internet, a conversation with the doctor, midwife or someone in your area. For example, it may have to do with nutrition, smoking, alcohol, a chronic illness or the use of medication. In the questionnaire we will use the term "healthy pregnancy".

Your participation
Participation in this study is entirely voluntary and without obligation. We ask you to complete a one-time questionnaire concerning your wishes and experiences. It takes approximately 10-15 minutes to complete this questionnaire. Healthcare providers will not see your answers. Completing the questionnaire also has no consequences for the care or treatment that you receive in the future. So you can freely give your opinion.

In addition, we request permission to see your pregnancy- and birth outcomes to link this to your answers to this questionnaire. We will only look at the outcomes of the pregnancy and delivery (for example: duration of pregnancy, method of delivery and weight of the child). If you give permission, we will receive this information from your midwife. You do not have to take any action yourself to provide this information.

Your information
The information that we collect will only be used for this scientific research. The data will be treated confidentially. That means that besides the research team nobody can see your pregnancy outcomes. Also in the final report, confidential information or personal information from or about you will not be disclosed in any way. The data will be kept protected in the Erasmus MC for a fixed period of 15 years.

Contact

For more information about the examination or questions about completing the questionnaire, please contact XX.

Participation

1. Do you want to participate in this study?

0 Yes

0 No

2. Which of these is your midwifery practice?

0 Amersfoort - Verloskundigenpraktijk de Kei

0 Barneveld - Verloskundigen Barneveld

0 Deventer - Verloskundigenpraktijk Anno

0 Deventer - Verloskundigenpraktijk Baren & Zo

0 Deventer - Verloskundigenpraktijk de Kuip

0 Tilburg - FAM

0 Utrecht - Verloskundigenpraktijk Doula

0 Zoetermeer - Embé Verloskundigen

0 Zoetermeer - Verloskundigenpraktijk Kéita

0 Zoetermeer - Verloskundigcentrum Partera

Part 1: Planning the pregnancy

3. How many times have you been pregnant, including this pregnancy?

___ times

4. How many weeks are you currently pregnant?

0 less than 12 weeks

0 more than 12 weeks

5. In the month that I became pregnant... (Please choose the statement which most applies to you):

0 I/we were not using contraception

0 I/we were using contraception, but not on every occasion

0 I/we always used contraception, but knew that the method had failed (i.e. broke, moved, came off, came out, not worked etc) at least once

0 I/we always used contraception

6. In terms of becoming a mother (first time or again), I feel that my pregnancy happened at the... (Please tick the statement which most applies to you):

0 Right time

0 Fine, but not quite right time

0 Wrong time

7. Just before I became pregnant... (Please tick the statement which most applies to you):

0 I intended to get pregnant

0 My intentions kept changing

0 I did not intend to get pregnant

8. Just before I became pregnant... (Please tick the statement which most applies to you)

0 I wanted to have a baby

0 I had mixed feelings about having a baby

0 I did not want to have a baby

9. Before I became pregnant.. (Please tick the statement which most applies to you)

0 My partner and I had agreed that we would like me to be pregnant

0 My partner and I had discussed having children together, but hadn’t agreed for me to get pregnant

0 We never discussed having children together

10. To what extent is the current pregnancy a deliberate choice?

No deliberate choice at all 1 2 3 4 5 Very deliberate choice

(If you choose 1 -> go to question 13)

11. How long did it take before you became pregnant?

__years __months

12. Did the current pregnancy occur spontaneously?

0 Yes

0 No, we used a fertility treatment (IVF / ICSI / etc.)

13. Below are a number of statements.

Indicate to what extent you agree with these statements.

- A pregnancy is not something you can plan, it is something that happens.
- I felt it was important to keep our wish to conceive private between me and my partner.
- I felt comfortable discussing our wish to conceive with friends and/or family.
- When we started trying to conceive, we thought "we'll see what happens".
- I thought it was romantic and / or intimate when we were trying to conceive a child.

Strongly disagree 1 2 3 4 5 Strongly agree

Part 2: Preparation for pregnancy

14. Did you search for or receive any information about a healthy pregnancy prior to your pregnancy?

0 Yes

0 No -> go to question 15

0 I don't remember -> go to question 15

15. How did you search for or receive this information? (multiple answers possible)

0 Internet

0 Books, magazines and / or folders-> go to question 17

0 Through family, acquaintances and / or friends -> go to question 17

0 Other, namely__________________________________________________________ -> go to question 17

16. Which of the following sites did you visit? (multiple answers possible)

0 www.zwangerwijzer.nl

0 www.slimmerzwanger.nl

0 www.strakszwangerworden.nl

0 www.nunietzwanger.nl

0 www.nietofwelzwanger.nl

0 www.12maandenzwanger.nl

0 www.24baby.nl

0 www.oudersvannu.nl

0 different, namely _____________________________________________________________________________

17. What was the main reason for looking up or receiving this information? (multiple answers possible)

0 I wanted to be well prepared for the pregnancy -> go to question 19

0 I did not get pregnant as quickly as hoped -> go to question 19

0 I felt insecure -> go to question 19

0 I had a (chronic) disease, disorder or health complication -> go to question 19

0 I had questions about my wish to conceive

0 Other, namely_______________________________________________________ -> go to question 19

18. I had questions about my wish to conceive, namely

______________________________________

19. Have you been in contact with a healthcare provider about your wish to conceive?

0 Yes

0 No -> go to question 24

0 I don't remember -> go to question 24

20. To which healthcare provider did you speak about your wish to conceive?

(multiple answers possible)

0 General practitioner

0 Midwife

0 Practical nurse

0 Gynecologist

0 Heredity expert

0 Other, namely_____________________________

21. Which topics have been discussed during your conversation with a healthcare provider? (multiple answers possible)

0 Healthy nutrition

0 Folic acid use

0 Smoking, alcohol, drugs, etc.

0 Fertility

0 Physical Activity / Exercising

0 Medication use

0 Infectious diseases and vaccinations

0 Working conditions

0 Treatment of chronic diseases

0 The previous pregnancy

0 Hereditary disorders

0 Other, namely_______________________

22. How useful did you find the information you received during this conservation?

Not useful at all 1 2 3 4 5 Very useful

23. How satisfied were you with the healthcare provider with whom you had this conversation?

Not at all satisfied 1 2 3 4 5 Very satisfied

-> go to question 27

24. Did you know that there was the possibility to talk to a healthcare provider about your wish to conceive?

0 Yes

0 No -> go to question 26

0 I don't remember -> go to question 26

25. What was the reason you did not opt for a conversation with a healthcare provider

about your wish to conceive? (multiple answers possible)

0 I already knew enough about a healthy pregnancy

0 I was pregnant faster than I thought

0 I didn't have time

0 I was not willing to pay for it

0 I did not know which healthcare provider I could go to with my wish to conceive

0 I did not like to talk freely about my wish to conceive

0 My wish to conceive was private and I would rather not share it with others

0 My partner could not come with

0 Other namely_____________________________________________________________________________________

26. If you look back on it now, how much need did you have for a conversation with a healthcare provider about your wish to conceive?

Not at all needed 1 2 3 4 5 Very much needed

*These questions (27 – 32) are only included in the questionnaire in the intervention period*

27. In your region we started an advertising campaign with among others these posters. Do you recognize these posters?

0 Yes

0 No -> go to question 31

0 I don't remember -> go to question 31

28. What do you remember most about the advertising campaign? (multiple answers possible)

0 The posters

0 Roadside signs

0 Stickers at the pharmacy

0 I don't know remember

0 Other, namely ______________________________________________________________________________

29. Did you look up information about a healthy pregnancy as a result of the advertising campaign?

0 Yes

0 No

0 I don't remember

30. To what extent did you feel that the message on the posters applied to you?

Didn't apply at all 1 2 3 4 5 Very much applied

31. Have you spoken to someone in your surroundings about the possibilities of becoming pregnant as a result of the advertising campaign?

0 Yes

0 No -> go to question 33

0 I don't remember -> go to question 33

32. Who did you talk to about this? (multiple answers possible)

0 Your partner

0 Friends

0 Family members

0 A healthcare provider

0 Other, namely ______________________________________________________________________________

33. Below are a number of statements about preparation for pregnancy. Indicate to what extent you agree with this statement.

- A good preparation for a pregnancy is important for every woman.
- A good preparation for a pregnancy is especially important during the first pregnancy.
- I knew where I could find information to prepare for this pregnancy.
- I believe I am healthy enough myself, so I didn't need any information about becoming pregnant in a healthy way.
- I believe that I could have influenced the chances for the future of our child by living healthier before pregnancy.
- I believe that the health of my partner before the pregnancy influences the future health of our child.
- I believe that my health during the pregnancy influences the future health of our child.
- I believe it is stressful to look up information about a (healthy) pregnancy.
- There are too many rules about what is and is not healthy during pregnancy.

Strongly disagree 1 2 3 4 5 Strongly agree

34. On a scale of 1 - 10, how well prepared did you feel for your current pregnancy?

Not at all prepared 1 2 3 4 5 6 7 8 9 10 Very well prepared

Part 3 Healthy lifestyle and risk factors

35. Below are a number of statements about preparation for pregnancy.

Indicate to what extent you agree with this statement.

- I think it is important to eat healthy before pregnancy
- I think it is important to eat healthy during pregnancy
- I think it is important not to smoke before the pregnancy
- I think it is important not to smoke during pregnancy
- I think it is important not to drink alcohol before pregnancy
- I think it is important not to drink alcohol during pregnancy

Strongly disagree 1 2 3 4 5 Strongly agree

36. How many pieces of fruit did you eat on average a day in the 3-month period before you knew you were pregnant? (a portion of small fruit equals one piece of fruit)

0 I never ate fruit

0 Less than 1 piece of fruit a day

0 1 piece of fruit a day

0 1-2 pieces of fruit a day

0 2 pieces of fruit a day

0 More than 2 pieces of fruit a day

37. How many grams of vegetables did you eat on average a day in the 3-month period before you knew you were pregnant? (one serving spoon of vegetables equals 50 grams)

0 I never ate vegetables

0 Less than 50 grams a day

0 50-100 grams a day

0 100-150 grams a day

0 150-200 grams a day

0 200 - 250 grams a day

0 more than 250 grams a day

38. How many glasses a day did you drink a caffeine-containing beverage on average in the 3-month period before you knew you were pregnant? (e.g. coffee, tea or cola)

___ glasses a day

39. Did you participate in Ramadan in the 3-month period before you knew you were pregnant?

0 Yes

0 No.

0 I don't know

40. On a scale of 1 -10, how would you describe your eating pattern in the 3-month period before you knew you were pregnant?

Very unhealthy 1 2 3 4 5 6 7 8 9 10 Very healthy

41. On a scale of 1 -10, how would you describe your eating pattern at the moment?

Very unhealthy 1 2 3 4 5 6 7 8 9 10 Very healthy

42. Which exercise activities did you do on average during the 3-month period before you knew you were pregnant? (multiple answers possible)

0 None

0 Slightly intensive exercise (eg cleaning, slow walking)

0 Moderately intensive exercise (eg cycling and yoga)

0 Heavy intensive exercise (eg running, cycling, soccer)

Average number of times per week:

Average number of hours per week (in total):

43. Which exercise activities are you currently doing?

0 None

0 Slightly intensive exercise (eg cleaning, slow walking)

0 Moderately intensive exercise (eg cycling and yoga)

0 Heavy intensive exercise (eg running, cycling, soccer

Average number of times per week:

Average number of hours per week (in total):

44. Did you smoke in the 3-month period before you knew you were pregnant?

0 Yes

0 No -> go to question 48

45. How many cigarettes did you smoke on average a day in the 3-month period before you knew you were pregnant?

 ___ cigarettes a day

46. Are you currently still smoking?

0 Yes

0 No, I quit before I became pregnant

0 No, I quit when I knew I was pregnant

0 No, I stopped during the first weeks of pregnancy

47. How many cigarettes did you smoke on average a day at the moment?

___ cigarettes a day

48. Is someone else in your household currently smoking?

0 Yes

0 No -> go to question 50

0 I don't remember -> go to question 50

49. What do you think about the fact that someone is smoking in your household?

Not at all disturbing 1 2 3 4 5 Very disturbing

50. Did you ever drink alcohol in the 3-month period before you knew you were pregnant?

0 Yes

0 No -> go to question 54

0 I don't remember -> go to question 54

51. ​​How many glasses of alcohol did you drink on average a week in the 3-month period before you knew you were pregnant?

___ glasses of alcohol per week

52. Do you currently still drink a glass of alcohol now and then?

0 Yes

0 No, I quit before I became pregnant -> go to question 54

0 No, I quit when I knew I was pregnant -> go to question 54

0 No, I stopped during the first weeks of pregnancy -> go to question 54

53. How many glasses of alcohol do you currently drink on average a week?

___ glasses of alcohol per week

54. Are you currently using folic acid? (Folic acid may also be included in multivitamins for pregnant women)

0 Yes

0 No --> go to question 58

55. Since when do you use folic acid?

0 More than 4 weeks before I knew I was pregnant

0 Less than 4 weeks before I knew I was pregnant

0 From the positive pregnancy test onwards

0 From the first few weeks of pregnant onwards

56. How many weeks were you pregnant when you started taking folic acid?

__ weeks pregnant

57. Why did you start using folic acid? (multiple answers possible)

0 I have heard about this from the midwife

0 I have heard about this from the general practitioner

0 I have heard about this from another healthcare provider

0 I have heard about this from family and / or friends

0 I have read about this in a book, magazine and / or folder

0 I have read about this on the internet

0 I knew this from my previous pregnancy

0 I just knew this

0 Other, namely__________________________

58. Do you have a chronic illness?

(For example: asthma, diabetes, cardiovascular disease or gastrointestinal and liver diseases)

0 Yes

0 No -> go to question 64

0 I don't know -> go to question 64

59. If yes, which chronic illness(es) do you have?

0 Asthma or another lung disease for which you also receive medication

0 Autoimmune disease

0 Blood coagulation problems

0 Cystic fibrosis (CF)

0 Epilepsy

0 Heart disease

0 High blood pressure

0 Cancer

0 Stomach, intestinal, pancreas and liver disease

0 Multiple sclerosis

0 Kidney and bladder disease

0 PKU

0 Psychiatric diseases

0 Rheumatism

0 Thyroid disease

0 Sickle cell disease

0 Diabetes

0 Thalassemia

0 Thrombosis or thrombosis bone

0 Pulmonary embolism

0 Stroke (TIA / CVA)

0 Tropical disease (eg malaria)

0 Other chronic, hereditary or congenital disease, namely_________________

60. Did you use prescription medication for your chronic illness in the 3-month period before you knew you were pregnant?

0 Yes

0 No -> go to question 64

0 I don't remember -> go to question 64

61. Has the dose of your medication been altered because of your pregnancy?

0 Yes

0 No -> go to question 64

0 I don't remember -> go to question 64

62. Since when has the dose of your medication been adjusted?

0 More than 4 weeks before I knew I was pregnant

0 Less than 4 weeks before I knew I was pregnant

0 From the positive pregnancy test onwards

0 From the first few weeks of the pregnancy onwards

63.How many weeks were you pregnant when the dosage of the medication was changed?

______ weeks pregnant

64. How often during your pregnancy have you used medication available without prescription? (For example: paracetamol, ibuprofen, allergy pills, etc.)

0 Daily

0 Few times a week

0 Monthly

0 Never

0 I don't remember

65. During the 3-month period before you knew you were pregnant, How often do you have negative feelings such as blue mood, despair, anxiety or depression?

0 Never

0 Rarely

0 Sometimes

0 Often

0 I don't remember -> go to question 67

66. Have these feelings changed significantly since you were pregnant?

0 Yes, these feelings have increased in quantity

0 Yes, these feelings have decreased in quantity

0 No.

0 I don't remember

0 Other, namely_________________________________________________________________________________________

67. How much social support from, for example, your partner, family, acquaintances, friends, neighbors, etc. are you currently experiencing?

Very little social support 1 2 3 4 5 Very much social support

68. Have you received all the vaccinations from your national vaccination program?

0 Yes

0 No.

0 I don't know

69. Which of the following working conditions apply to you? (multiple answers possible)

0 I work in shifts

0 For my job I regularly have to do heavy physical work, stand for a long time and / or walk a lot

0 I come into contact with sick people

0 I come into contact with animals

0 I come into contact with small children

0 I come into contact with nature in the woods or park

0 I come in contact with raw meat

0 I come in contact with (cheese from) raw milk

0 I come into contact with unwashed raw vegetables / fruit

0 I come in contact with liver products (or something else with a lot of vitamin A)

0 I come into contact with waste or waste water

0 I come in contact with blood, urine, saliva or other body fluids

0 I come in contact with stools

0 I come into contact with radioactive radiation

0 I come in contact with chemical substances

0 None of the above applies to me.

70. Are there any hereditary defects or diseases in your family or in your partner's family?
(Also consider the relatives who have died or stillborn children in your family who may have been sick Or relatives who have had several miscarriages)

0 Yes

0 I don't remember -> go to question 74

0 No -> go to question 74

71. Which hereditary defects or diseases occur in your family or in the family of your partner? (multiple answers possible)

0 Blindness or severe visual impairment

0 Down's syndrome

0 Epilepsy, seizures, (fever) convulsions

0 Severe deafness

0 Congenital heart defects

0 Cardiac arrhythmias

0 Heart attack before 35^th^ year

0 Hemophilia or carrier of hemophilia

0 Disorders of the brain, nervous system or muscles

0 Lip or palate cleft

0 Spina bifida, open skull (anencephaly), hydrocephalus.

0 Sickle cell disease or carrier of sickle cell disease (hereditary anemia)

0 Muscle diseases (eg Duchenne's disease)

0 Cystic fibrosis or carrier of CF

0 Thalassemia or carrier of thalassemia (hereditary anemia)

0 Two or more miscarriages in one person

0 Delayed intellectual development (eg, fragile X syndrome)

0 Other hereditary disorder namely ___________________________________________________________

72. Did you learn about the possible risks of this hereditary defect before you knew you were pregnant?

0 Yes

0 No -> go to question 74

0 I don't know -> go to question 74

73. In what way did you learn about the possible risks of this hereditary defect(s)?

0 Through the Internet

0 Through Books / Magazines / Folders

0 Through a healthcare provider

0 Through Family / Acquaintances / Friends

0 Through your Partner

0 Otherwise, namely _______________________________________________________________________________

74. On a scale of 1 to 10, how prepared do you currently feel about parenthood?

Not at all prepared 1 2 3 4 5 6 7 8 9 10 Very prepared

Part 4: Your personal situation

75. Have you ever had a miscarriage in the past?

0 Yes

0 No -> go to question 77

0 I don't know -> go to question 77

76. How many times have you had a miscarriage in the past?

___ times

77. Have you ever given birth before?

0 Yes

0 No -> go to question 81

78. How often did you give birth?

0 Yes

0 No

79. Have you ever given birth to a child with a birth defect? (For example a club foot, cleft lip, Down's syndrome).

0 Yes

0 No -> go to question 81

80. My child has the following birth defect:

0 Blindness or severe visual impairment

0 Down's syndrome

0 Epilepsy, seizures, (fever) convulsions

0 Severe deafness

0 Congenital heart defects

0 Cardiac arrhythmias

0 Hemophilia or carrier of hemophilia

0 Disorders of the brain, nervous system or muscles

0 Lip or palate cleft

0 Spina bifida, open skull (anencephaly), hydrocephalus.

0 Sickle cell disease or carrier of sickle cell disease (hereditary anemia)

0 Muscle diseases (eg Duchenne's disease)

0 Cystic fibrosis or carrier of CF

0 Thalassemia or carrier of thalassemia (hereditary anemia)

0 Delayed intellectual development (eg, fragile X syndrome)

0 Other hereditary disorder namely ___________________________________________________________

81. What is your age? _____ years old

82. What is your height in centimeters? ____ cm

83. What was your weight prior to pregnancy? ____ kg

84. What are the four numbers of your zip code? _____

85. What is your highest level of education?

0 none (yet)

0 primary school

0 primary vocational education

0 MAVO / VMBO

0 HAVO

0 VWO / athenaeum / gymnasium

0 MBO

0 Applied sciences (HBO)

0 University

0 Other, namely___________________

86. Are you currently studying?

0 Yes

0 No -> go to question 88

87. Yes, namely the following education:

0 Primary vocational education

0 MAVO / VMBO

0 HAVO

0 VWO / atheneum / gymnasium

0 MBO

0 Applied Sciences (HBO)

0 University

0 Other, namely _________________________

88. What is your monthly net family income? (from you and your partner together)

0 Up to € 1,600

0 € 1,600 - € 2,400

0 € 2,400 - € 3,500

0 Above € 3,500

0 I don't remember

0 I prefer not to answer this question

89. Do you sometimes have trouble making ends meet with your / your family’s income?

0 Always

0 Many months of the year

0 Some months of the year

0 Never

0 I don't know

0 I prefer not to answer this question

90. What is your current family situation?

0 Single

0 Married

0 Living together with partner

0 Divorced / widowed

0 Living with parents

0 Other, namely ____________________

91. Are you currently working a paid job?

0 Yes

0 No -> go to question 93

92. How many hours a week do you work a paid job?

____ hours per week -> go to question 94

93. What do you do in daily life? (multiple answers possible)

0 Care duties e.g. for children or family)

0 Volunteering

0 Full-time study

0 Part-time study

0 Sickness benefits act

0 Other, namely______________________________________________________________________________________

94. To which of the following ethnical backgrounds do you identify yourself with most?

0 Dutch

0 Turkish

0 Moroccan

0 Indonesian

0 German

0 Surinamese

0 Polish

0 Belgian

0 Other, namely_____________________

0 I don't remember

Part 5: Father of the baby

95. To which of the following ethnical backgrounds does the father of your baby identify the most?

0 Dutch

0 Turkish

0 Moroccan

0 Indonesian

0 German

0 Surinamese

0 Polish

0 Belgian

0 Other, namely_____________________

0 I don't know

96. What is your current relationship with your baby's father?

0 Married

0 Cohabiting

0 Relationship, but not cohabiting

0 Father is no longer in the picture -> go to question 107

0 Father is unknown -> go to question 107

From this point on, the father of the baby will be referred to as your partner.

97. Are you (distant) family of your partner?

0 Yes

0 No

0 I don't remember

98. How would you describe your partner's lifestyle at the moment?

Very unhealthy 1 2 3 4 5 Very healthy

99. Does your partner smoke?

0 Yes

0 Not anymore, he stopped smoking before you knew you were pregnant

0 Not anymore, he stopped smoking during the pregnancy

0 No

100. How many cigarettes does your partner smoke on average per day?

___ cigarettes a day

101. Does your partner ever drink alcohol?

0 Yes

0 Not anymore, he stopped drinking alcohol before you knew you were pregnant

0 Not anymore, he stopped drinking alcohol during the pregnancy

0 No

102. How many glasses of alcohol does your partner drink on average per week?

___ glasses per day

103. How important do you think it is that your partner lives a healthy lifestyle in the period before pregnancy?

Not important at all 1 2 3 4 5 Very important.

104. On a scale of 1 to 10, how involved was your partner when you were preparing for the pregnancy?

Not involved at all 1 2 3 4 5 6 7 8 9 10 Very involved

105. On a scale of 1 to 10, how involved is your partner in your current pregnancy?

Not involved at all 1 2 3 4 5 6 7 8 9 10 Very involved

106. We would also like your partner to complete a short questionnaire.

Do you want to give us his e-mail address in this field?

_______________________________________________________________

Pregnancy outcomes

To investigate whether a good preparation for the pregnancy also leads to better health outcomes for mother and child, we would like to follow-up on your pregnancy- and birth outcomes. This way we can link your pregnancy outcomes to your answers to this questionnaire. We will only look at the outcomes of the pregnancy and birth (for example: duration of pregnancy, method of delivery and weight of the child). If you give permission, we will receive this information from your midwife. You do not have to take any action yourself to provide this information.

The information that we collect will only be used for this scientific research. Your information will be treated confidentially. That means that besides the research team nobody can see your information. Also in the report no confidential or personal information will be disclosed in any way. Your information will be kept protected in the Erasmus MC for a fixed period of 15 years.

107. I want to give permission to follow-up on my pregnancy outcomes after giving birth. (If you wish to give your consent, you will first receive detailed information regarding the use of your personal data due to the AVG legislation)

0 Yes

0 No.

You have reached the end of this questionnaire. Thank you so much for participating. Your opinion and experiences are greatly appreciated. Your data will be treated confidentially. If you have any questions, please contact us via XX.
