# Supplementary material for: The effect of a locally tailored intervention on the uptake of preconception care in the Netherlands: a stepped-wedge cluster randomized trial (APROPOS-II study)
Source: BMC Public Health. 2022 Nov 1;22:1997. doi: 10.1186/s12889-022-14343-x (PMC9623982; doi:10.1186/s12889-022-14343-x)
Supplement: Supplementary file 1 — Additional file 1: Supplemental File 1. Questionnaire for women. Supplemental File 2. Questionnaire for men. Supplemental File 3. Questionnaire for healthcare providers. [file 12889_2022_14343_MOESM1_ESM.zip › 20220622 - Supplemental file 2 - Questionnaire men.docx]

APROPOS-II Questionnaire Prospective Father

Will you help improve health care in the period before pregnancy?

APROPOS-II Project

For a scientific study from Erasmus MC in Rotterdam we want to know how prospective parents prepare for the pregnancy and what these preparations have for consequences on the health of mothers and their babies. Your opinion and experiences are very important for us. Through a questionnaire we can investigate what your wishes, needs and experiences are regarding a healthy pregnancy. Through your participation we can improve the care for women who want to become pregnant in the future.

Good preparation for a pregnancy

You can think of reliable information on the internet or a conversation with the doctor, midwife or someone in your area. For example, it may have to do with food, smoking, alcohol, chronic illness or the use of medication. It is also possible to go to a children's wish consultation hour. There you can discuss with a healthcare provider what you can do to become pregnant as healthy as possible.

Your participation

Participation in this study is entirely voluntary and without obligation. We ask you to complete a one-time questionnaire concerning your wishes and experiences. It takes approximately 15 minutes to complete this questionnaire. Healthcare providers will not see your answers. Completing the questionnaire also has no consequences for the care or treatment that you receive in the future. So you can freely give your opinion.

Your information

The information that we collect will only be used for this scientific research. The data will be treated confidentially. That means that besides the research team nobody can see your pregnancy outcomes. Also in the final report, confidential information or personal information from or about you will not be disclosed in any way. The data will be kept protected in the Erasmus MC for a fixed period of 15 years.

Contact

For more information about the examination or questions about completing the questionnaire, please contact XX.

Participation

1. Do you wish to participate in this study

0 Yes

0 No

Part 1: Wish to conceive

2. Did you discuss with your partner prior to the pregnancy whether the both of you were ready for children?
0 Yes
0 No
0 I don't know

3. Below are a number of statements. Indicate to what extent you agree with these statements.

- It is up to the woman to initiate talking about having a children.
- A pregnancy is not something you can plan, it is something that happens spontaneously.
- The current pregnancy was planned.
- I thought it was important to keep our wish to conceive private between me and my partner.
- I felt comfortable discussing our wish to conceive with friends or family.
- When we started trying to conceive, we thought "we'll see what happens".
- I thought it was romantic and / or intimate when we were trying to conceive a child.
- I think this pregnancy is taking place at the right time in our lives.

Strongly disagree 1 2 3 4 5 Strongly agree

Part 2: Preparation for pregnancy

4. Did you search for or receive any information about a healthy pregnancy prior to the pregnancy?

0 Yes

0 No -> go to question 9

0 I don't remember -> go to question 9

5. How did you search for or receive this information? (multiple answers possible)

0 Through the Internet

0 Through books, magazines and / or folders -> go to question 7

0 Through family, acquaintances and / or friends -> go to question 7

0 Through my partner -> go to question 9

0 Other, namely_____________________________________________________________ -> go to question 7

6. Which of the following sites did you visit?

0 www.zwangerwijzer.nl

0 www.slimmerzwanger.nl

0 www.strakszwangerworden.nl

0 www.nunietzwanger.nl

0 www.nietofwelzwanger.nl

0 www.12maandenzwanger.nl

0 www.24baby.nl

0 www.oudersvannu.nl

0 different, namely _____________________________________________________________________________

7. What was the main reason for looking up or receiving this information? (multiple answers possible)

0 I wanted to be well prepared for the pregnancy -> go to question 9

0 My partner recommended it to me -> go to question 9

0 We did not get pregnant as quickly as hoped -> go to question 9

0 I felt insecure -> go to question 9

0 I had a (chronic) disease, disorder or health complication -> go to question 9

0 I had questions about my wish to conceive

0 Other, namely________________________________________ -> go to question 9

8. I had a question about my wish to conceive, namely

_____________________________________________

9. Have you been in contact with a healthcare provider about your wish to conceive?

0 Yes

0 No -> go to question 11

0 I don't remember -> go to question 11

10. How useful was the information you received during this conservation?

Not useful at all 1 2 3 4 5 Very useful

-> go to question 14

11. Did you know that there was the possibility to talk to a healthcare provider about your wish to conceive?

0 Yes

0 No -> go to question 13

0 I don't remember -> go to question 13

12. What was the reason you did not opt for a conversation with a healthcare provider about your wish to conceive? (multiple answers possible)

0 I already knew enough about a healthy pregnancy

0 I didn't need a conversation with a healthcare provider

0 My partner was pregnant faster than expected

0 I didn't have time

0 I was not willing to pay for it

0 I did not know which healthcare provider I could go to with my wish to conceive

0 I did not like to talk freely about my wish to conceive

0 My wish to conceive was private and I would rather not share it with others

0 Other namely_____________________________________________________________________________________

13. If you look back on it now, how much did you need a conversation with a healthcare provider about your wish to conceive?

Not at all needed 1 2 3 4 5 Very much needed

14. Are there any hereditary defects or diseases in your family or in your partner's family? (Also consider the relatives who have died or stillborn children in your family who may have been sick. Or relatives who have had several miscarriages.)

0 Yes

0 No -> go to question 18

0 I don’t remember -> go to question 18

15. Which hereditary defects or diseases occur in your family or in the family of your partner? (multiple answers possible)

• Blindness or severe visual impairment

• Down's syndrome

• Epilepsy, seizures, (fever) convulsions

• Severe deafness

• Congenital heart defects

• Cardiac arrhythmias

• Heart attack before 35^th^ year

• Hemophilia or carrier of hemophilia

• Disorders of the brain, nervous system or muscles

• Lip or palate cleft

• Spina bifida, open skull (anencephaly), hydrocephalus.

• Sickle cell disease or carrier of sickle cell disease (hereditary anemia)

• Muscle diseases (eg Duchenne's disease)

• Cystic fibrosis or carrier of CF

• Thalassemia or carrier of thalassemia (hereditary anemia)

• Two or more miscarriages in one person

• Delayed intellectual development (eg, fragile X syndrome)

• Other hereditary disorder namely ___________________________________________________________

16. Did you learn about the possible risks of this hereditary defect before you knew you were pregnant?

0 Yes

0 No -> go to question 18

0 I don’t remember -> go to question 18

17. In what way did you learn about the possible risks of this hereditary defect?

0 Through the internet

0 Through books / magazines / folders

0 Through a healthcare provider

0 Through family / acquaintances / friends

0 Through your partner

0 Otherwise, namely _______________________________________________________________________________

*These questions (18 – 23) are only included in the questionnaire in the intervention period*

18. In your region we started an advertising campaign with among others these posters. Do you recognize these posters?

0 Yes

0 No -> go to question 22

0 I don't remember -> go to question 22

19. What do you remember most about the advertising campaign? (multiple answers possible)

0 The posters

0 Roadside signs

0 Stickers at the pharmacy

0 I don't know remember

0 Other, namely ______________________________________________________________________________

20. Did you look up information about a healthy pregnancy as a result of the advertising campaign?

0 Yes

0 No.

0 I don't remember

21. To what extent did you feel that the message on the posters applied to you?

Didn't apply at all 1 2 3 4 5 Very much applied

22. Have you spoken to someone in your surroundings about the possibilities of preparing for the pregnancy as a result of the advertising campaign?

0 Yes

0 No -> go to question 24

0 I don't remember -> go to question 24

23. Who did you talk to about this? (multiple answers possible)

0 Your partner

0 Friends

0 Family members

0 A healthcare provider

0 Other, namely ______________________________________________________________________________

24. Below are a number of statements about preparation for pregnancy. Indicate to what extent you agree with this statement.

0 I was well prepared for this pregnancy.

0 I knew where I could find information to prepare for this pregnancy.

0 I was involved in the preparations for this pregnancy.

0 I feel that as a man I can contribute to a healthy pregnancy.

0 I feel that as a man I can influence the chances for the future of my child by living a healthy lifestyle.

0 I feel that the health of my partner before pregnancy affects the future health of our child.

0 When a prospective father smokes or drinks alcohol in the period before the pregnancy, this can affect the quality of his sperm.

0 I believe it is stressful to look up information about a (healthy) pregnancy.

0 There are too many rules about what is and is not healthy during pregnancy.

0 I feel involved in this pregnancy.

25. Would you have liked to be more involved during the preparation for the pregnancy?

0 Yes

0 No -> go to question 27

0 I don’t remember -> go to question 27

26. Do you have a suggestion about how you could have been more involved during the preparation for the pregnancy?

Part 3: Lifestyle

27. On a scale of 1 - 10, how healthy would you describe your lifestyle in the 3-month period before the pregnancy?

Very unhealthy 1 2 3 4 5 6 7 8 9 10 Very healthy

28. How important do you think it is to adopt a healthy lifestyle in the 3-month period before the pregnancy?

Not important at all 1 2 3 4 5 Very important.

29. On a scale of 1 - 10, how healthy would you describe your lifestyle since pregnancy?

Very unhealthy 1 2 3 4 5 6 7 8 9 10 Very healthy

30. Do you smoke?

0 Yes

0 Not anymore, I stopped smoking before the pregnancy was known -> go to question 32

0 Not anymore, I stopped smoking after the pregnancy was known -> go to question 32

0 No -> go to question 32

31. How many cigarettes did you smoke on average a day?

___ cigarettes a day

32. Do you regularly drink alcohol?

0 Yes

0 Not anymore, I stopped smoking before the pregnancy was known -> go to question 34

0 Not anymore, I stopped smoking after the pregnancy was known -> go to question 34

0 No -> go to question 34

33. How many glasses of alcohol do you currently drink on average a week?

___ glasses of alcohol per week

Part 4: Personal information

34. What is your age? ___ years old

35. What is your highest level of education?

0 None (yet)

0 Primary school

0 Primary vocational education

0 MAVO / VMBO

0 3-Year HAVO

0 HAVO

0 VWO / athenaeum / gymnasium

0 MBO

0 Applied sciences (HBO)

0 University

0 Other, namely___________________

36. To which of the following ethnical backgrounds do you identify yourself with most?

0 Dutch

0 Turkish

0 Moroccan

0 Indonesian

0 German

0 Surinamese

0 Polish

0 Belgian

0 Other, namely_____________________

0 I don't remember

37. This is your .. time becoming a father __ time

Future research

You have reached the end of this questionnaire. To improve healthcare, we would also like to use your answers to these questions for future research in the field of preconception care. If you want to give permission, you will first receive detailed information before you actually give permission.

38. I want to give permission for the use of my answers to this questionnaire for future research in the field of preconception care. (If you wish to give your consent, you will first receive detailed information regarding the use of your personal data due to the AVG legislation)

0 Yes

0 No.

You have reached the end of this questionnaire. Thank you so much for participating. Your opinion and experiences are greatly appreciated. Your data will be treated confidentially. If you have any questions, please contact us via XX.
