# Supplementary material for: The effect of a locally tailored intervention on the uptake of preconception care in the Netherlands: a stepped-wedge cluster randomized trial (APROPOS-II study)
Source: BMC Public Health. 2022 Nov 1;22:1997. doi: 10.1186/s12889-022-14343-x (PMC9623982; doi:10.1186/s12889-022-14343-x)
Supplement: Supplementary file 1 — Additional file 1: Supplemental File 1. Questionnaire for women. Supplemental File 2. Questionnaire for men. Supplemental File 3. Questionnaire for healthcare providers. [file 12889_2022_14343_MOESM1_ESM.zip › 20220622 - Supplemental file 3 - Questionnaire healthcare providers.docx]

APROPOS-II Questionnaire healthcare providers

The purpose of this questionnaire is to learn more about the experiences of local healthcare providers with preconception care. The questionnaire is processed anonymously. The results of this questionnaire will be used for scientific research and for a locally tailored preconception care focused on regional needs and situations.

1. In which municipality do you work?

- Barneveld
- Deventer
- Zoetermeer
- Amersfoort

1. What is your occupation?
   - Midwife
   - Gynaecologist
   - General practitioner
   - Dietitian
   - Physiotherapist
   - Maternity care assistant
   - Pharmacist or pharmacy employee
   - Preventive child healthcare professional
   - Social worker
   - Nurse practitioner
   - Other, namely ________________________________________________________
2. How many years of working experience do you have in this function?

_________________

1. Do you provide PCC-consultations to women and/or men in the fertile phase of their life?

(A PCC-consultation was defined as providing information, educating prospective parents or determining individual policy about an upcoming pregnancy)

- Yes, namely ___ times per year
- No 🡪 continue to question 6
- Don’t remember 🡪 continue to question 6

5. At what kind of occasion did you provide a PCC-consultation?

- I personally approached a patient/client or gave advice.
- I answered a question from a patient/client
- At another occasion, namely _______

6. Below a number of statements are presented, to what extent do you agree with the following statements?

- Do you feel that providing PCC-information is currently a routine part of your job?
- Do you think that providing PCC-information could become a routine part of your job?
- When you provide PCC-information, how familiar does this feel?

Not at all 1 2 3 4 5 6 7 8 9 10 Completely

7. Below a number of statements are presented, to what extent do you agree with the following statements?

- I see the potential added value of PCC in my work
- I can easily integrate PCC into my current job
- Sufficient resources are available to support providing PCC
- I am aware of the recent literature/guidelines on the effects of PCC
- I appreciate the effects PCC had on my job
- I have enough time to provide PCC-consults
- I think PCC is well organised in my area of work

Completely disagree 1 2 3 4 5 Completely Agree

*These statements are only presented for healthcare providers other than midwives*

8. Below a number of statements are presented, to what extent do you agree with the following statements?

- I find it difficult to start a conversation about a (possible) wish to conceive
- I feel competent to provide PCC-information to couples with a wish to conceive
- I think it is part of my job to provide PCC-information to couples with a wish to conceive

Completely disagree 1 2 3 4 5 Completely Agree

Thank you for your cooperation!
